# Case Report: Need for Caution in the Diagnosis of GFAP Astrocytopathy—A Case of GFAP Astrocytopathy Coexistent With Primary Central Nervous System Lymphoma

**DOI:** 10.3389/fneur.2022.806224

**Published:** 2022-01-28

**Authors:** Jia Fang, Zhongyi Tong, Wei Lu

**Affiliations:** ^1^Department of Neurology, Second Xiangya Hospital, Central South University, Changsha, China; ^2^Department of Pathology, Second Xiangya Hospital, Central South University, Changsha, China

**Keywords:** case report, autoimmune GFAP astrocytopathy, lymphoma, brain biopsy, glial fibrillary acidic protein, steroid

## Abstract

We reported a case of primary central nervous system lymphoma (PCNSL) coexistent with glial fibrillary acidic protein (GFAP) astrocytopathy, and discussed the problems needing attention in the diagnosis and differential diagnosis of GFAP astrocytopathy. Our patient was a 51-year-old female who presented with somnolence for a month, and memory declination for 10 days. Brain magnetic resonance imaging (MRI) demonstrated multiple abnormal enhancement lesions in bilateral basal ganglia and around the third ventricle, as well as transient T2-weighted hyper-intensity lesions at the splenium of the corpus callosum during the course of the disease. The cerebrospinal fluid (CSF) was positive for anti-GFAP antibodies by antigen-transfected HEK293 cell-based assay (indirect immunofluorescence assay). She was initially diagnosed with autoimmune GFAP astrocytopathy. After treatment with corticosteroids for about 2 months, she displayed poor response and even worsened clinical manifestations when the dose of prednisone reduced to 45 mg. Stereotactic brain biopsy was adopted and the diagnosis of large B-cell lymphoma, non-germinal center type was established on pathological examination. The results of brain biopsy also showed perivascular inflammation and CD8+ T cell infiltration, which also accorded with GFAP astrocytopathy. After chemotherapy with rituximab and methotrexate, the patient showed clinical and radiological improvement significantly. Our findings suggest that positivity of GFAP antibody calls for cautious interpretation. Cancer screening appropriate for age, sex, and risk factors is recommended for GFAP antibody-positive patients, especially for patients with atypical clinical and radiologic manifestations.

## Introduction

Autoimmune glial fibrillary acidic protein (GFAP) astrocytopathy is a newly described entity of immunotherapy-responsive autoimmune inflammatory central nervous system diseases which is confirmed by specific GFAP-immunoglobulin G (IgG) in the cerebrospinal fluid (CSF) (1). Its clinical presentations include encephalopathy, headache, myelopathic symptoms, abnormal vision/optic neurities, postural tremor, and cerebellar ataxia (1). Brain magnetic resonance imaging (MRI) abnormalities are common. Lesions involved the subcortical white matter, basal ganglia, hypothalamus, brainstem, cerebellum, meninges, ventricle, and skull, often accompanied by a hallmark brain linear perivascular radial gadolinium enhancement on MRI (2).

However, whether GFAP antibody is a bystander or directly involved in pathogenesis still remains controversial (3). One-third of cases with positive GFAP-antibody have serologic evidence of autoimmune endocrinopathy; more than one-third are paraneoplastic (4). Therefore, it is recommended to screen for underlying neoplasms within 2 years of GFAP disease onset (5).

Herein, we described a case of autoimmune GFAP astrocytopathy coexistent with primary central nervous system lymphoma (PCNSL), and investigated common problems in diagnosis and differential diagnosis of autoimmune GFAP astrocytopathy.

## Case Presentation

**Figure 1:**
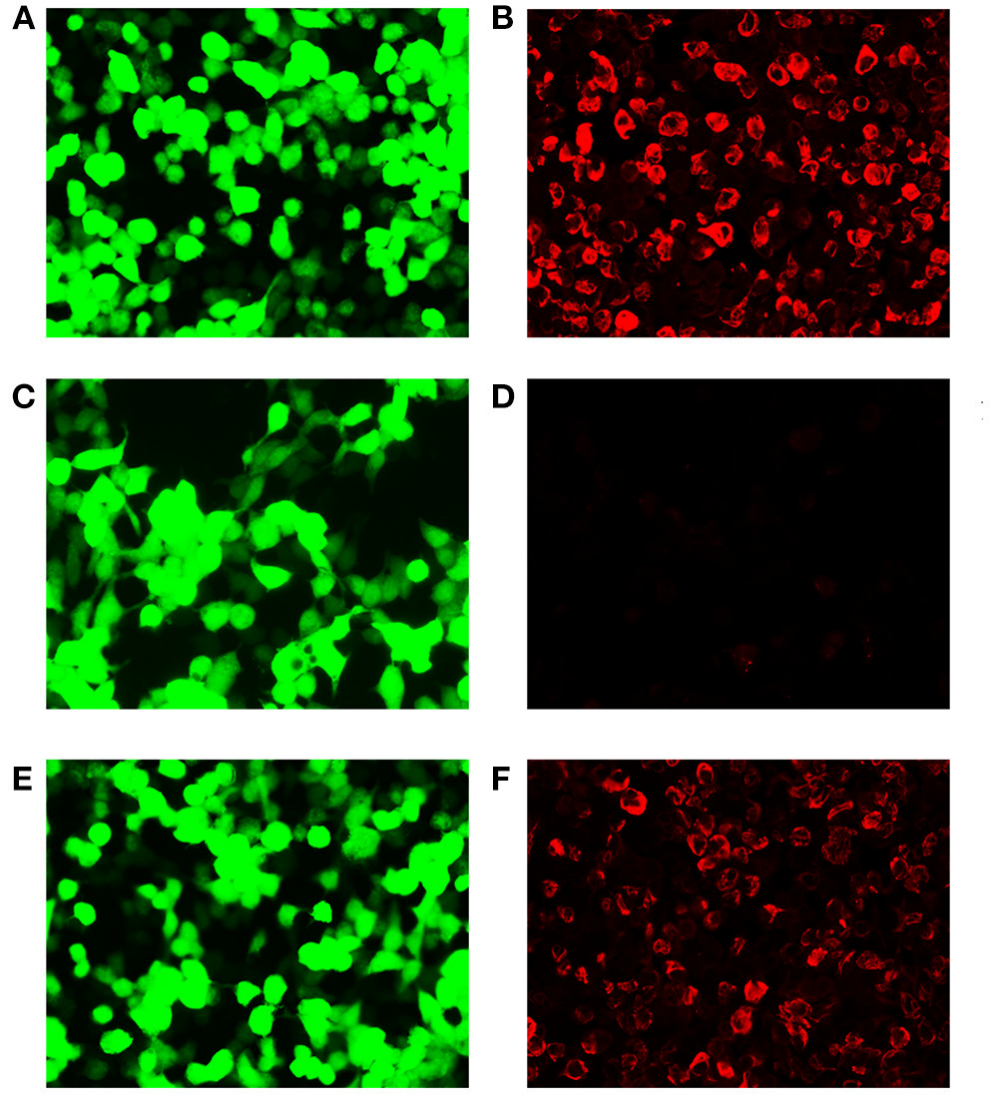
Demonstration of GFAP-IgG by GFAP-transfected HEK293 cell-based immunofluorescence assay. **(A,C,E)** HEK293 cells expressing green fluorescent protein (GFP)-tagged GFAP (green). **(B,D,F)** HEK293 cells immunostained (red if positive); **(B)** Positive control with human anti-GFAPα IgG. **(D)** Negative with healthy control. **(F)** Positive result of cerebrospinal fluid (CSF) (titer at 1:32). Scale bar = 50 μm.

**Figure 2:**
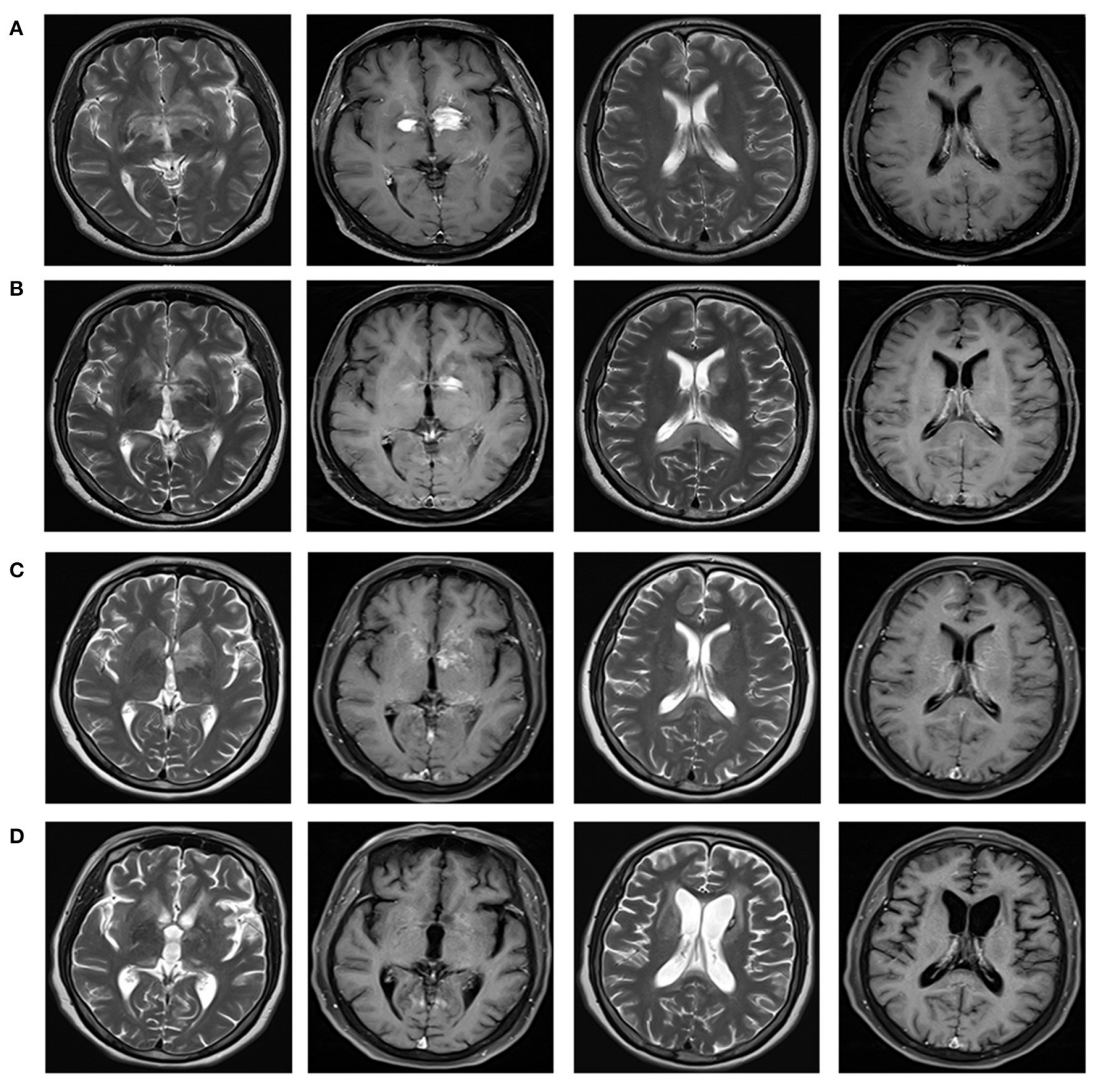
Brain magnetic resonance imaging reflecting the imaging changes of lesions in bilateral basal ganglia and around the third ventricle, and the changes of splenial corpus callosum lesions during the course of disease. Brain MRI with T2-weighted and T1 contrast-enhanced were shown in different columns. **(A)** In December 2020, multiple abnormal enhancement lesions in bilateral basal ganglia and around the third ventricle were demonstrated. **(B)** In January 2021, lesions in bilateral basal ganglia and around the third ventricle reduced significantly with less enhancement. A new T2 signal in the splenium of the corpus callosum without enhancement was shown. **(C)** In March 2021, lesions in bilateral basal ganglia and around the third ventricle were the same as before, with increased enhancement. Attenuated T2 signal abnormality in the splenium of the corpus callosum was observed. **(D)** In August 2021, lesions in the bilateral basal ganglia and around the third ventricle significantly reduced, with complete resolution of the enhanced lesions. Abnormal signal foci in the splenium of the corpus callosum disappeared.

On December 25, 2020, a 51-year-old Chinese female went to our hospital with chief complaints of somnolence for a month, and memory declination for 10 days. No previous history of infection or vaccination before disease onset was found. Her medical and family history was unremarkable. A weight loss of more than 10 kg was reported in the last month. Pertinent positive finding on physical examination at hospital admission included somnolence. Her cranial nerves, strength, and sensory examinations were normal. Antinuclear and antineutrophil cytoplasmic antibodies, human immunodeficiency virus, hepatitis B virus, hepatitis C virus, JC virus, and syphilis were negative. Chest X-ray, abdominal ultrasonography, and gynecological ultrasonography were normal. Lumbar puncture revealed a slightly elevated white blood cell count (12/mm^3^) with 83% monocytes and a nearly normal protein level of 0.455 g/l (normal range: 0.15–0.45 g/l). High titers of anti-GFAP IgG antibodies (1:32) were found in CSF but not in serum (Figure 1). No malignant cells were found in the CSF. Tests of other autoantibodies in CSF and serum, including MOG-IgG, MBP-IgG, AQP4-IgG, NMDAR-IgG, AMPA-IgG, LGI1-IgG, CASPR2-IgG, and GABABR-IgG were negative. Blood neoplastic and paraneoplastic markers were negative. Electroencephalogram indicated mild abnormality. FDG-PET scan suggested autoimmune or infectious lesions, however neoplasia cannot be excluded. There were patchy and nodular areas with increased glucose metabolism in bilateral basal ganglia and periventricular area. Magnetic resonant imaging (MRI) of brain (Figure 2A) showed multiple abnormal enhancement lesions in bilateral basal ganglia and around the third ventricle. Based on clinical data and CSF analysis, a diagnosis of GFAP astrocytopathy was established. The patient was treated with intravenous methylprednisone (1,000 mg for 3 days) followed by a 50% reduction of the dose after 3–5 days, and subsequently oral prednisone tablets (60 mg/day), which was then tapered (reduced 5 mg/day every 2 weeks). A repeated brain MRI (Figure 2B) scan showed a significant reduction in the former lesion size and a new T2 signal in the splenium of the corpus callosum without contrast enhancement. After discharge, the patient took prednisone to inhibit immunity. Her drowsiness and short-term memory has significantly improved. One month after discharge, the patient went to the outpatient clinic of our hospital for follow-up. A repeat CSF analysis was unremarkable. The GFAP antibody in CSF and serum was negative. She was admitted again for cognitive impairment and slow response when the dose of prednisone reduced to 45 mg. Positive physical examinations included somnolence, near memory loss and decreased computational ability. A rechecked lumbar puncture revealed 0 WBC/μl, glucose of 4.42 mmol/L, and normal protein level (451 mg/l). Serological tests for infectious, autoimmune, and neoplastic parameters were all normal. The GFAP antibody in CSF and serum was negative either. The brain MRI scan (Figure 2C) showed attenuated T2 signal abnormality in the splenium of the corpus callosum, but lesions in bilateral basal ganglia and around the third ventricle were the same as before, with increased enhancement, and new lesions in the right frontal lobe. PET-CT demonstrated enhanced glucose metabolism in the bilateral basal ganglia and periventricular area, with lesion size larger than before and there was new nodular increase of glucose metabolism under the right frontal cortex. Considering the poor response to steroid therapy, coexistence of malignancy was suspected and a stereotactic brain biopsy was adopted.

**Figure 3:**
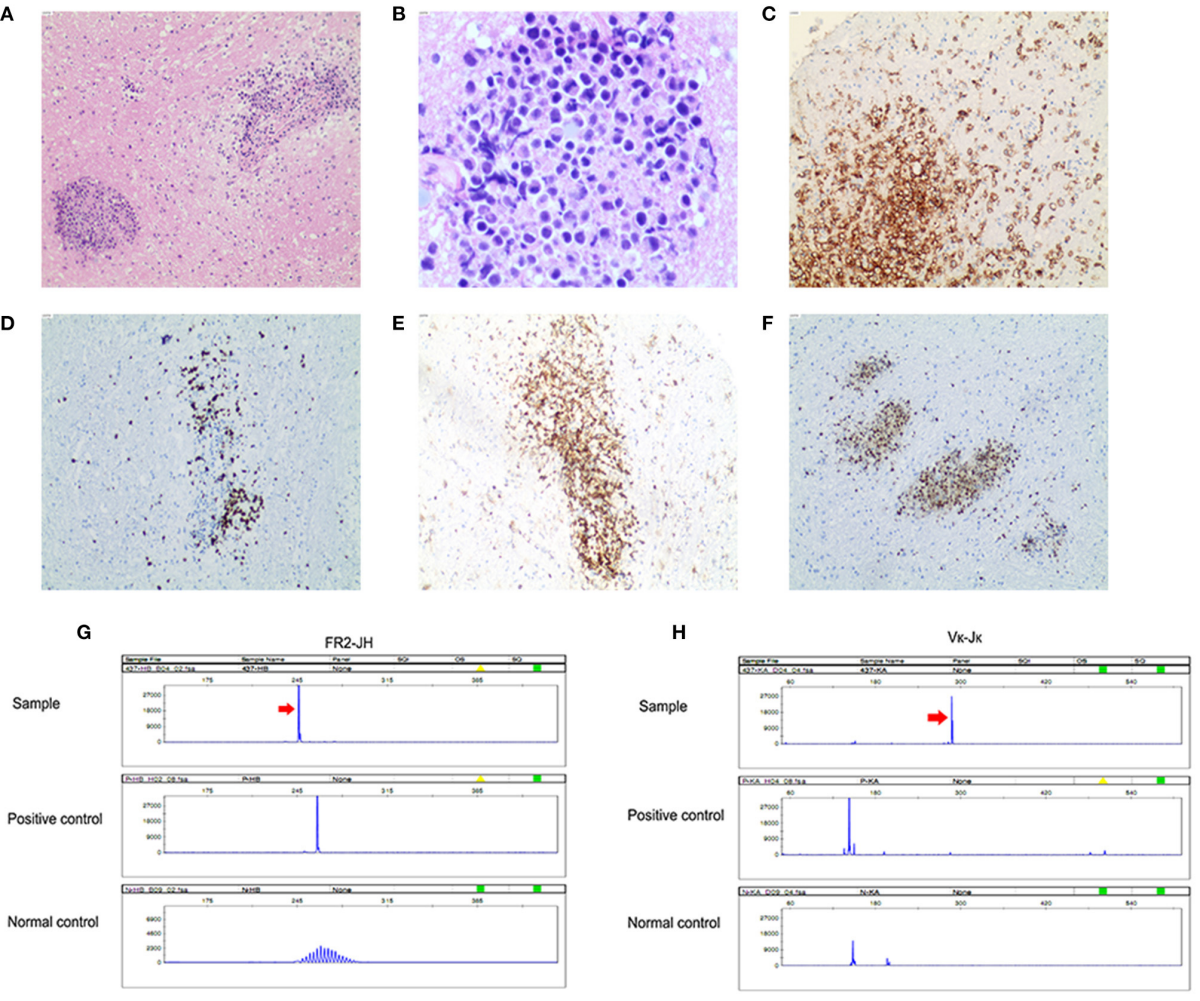
Primary central nervous system lymphoma (diffuse large B-cell lymphoma). **(A)** Angiocentric pattern of distribution of atypical lymphoid cells (original magnification ×100); **(B)** Atypical lymphoid cells are large with irregular nuclei and scant cytoplasm (original magnification ×400); **(C)** Tumor cells are diffusely positive for CD20 (original magnification ×100) and **(D)** usually show a high Ki-67 proliferation index (original magnification ×100); **(E)** Tumor cells are positive for BCL 2 (original magnification ×100); **(F)** Tumor cells are positive for MUM-1 (original magnification ×100). PCR analysis of B-cell monoclonal immunoglobulin heavy chain gene rearrangement detected monoclonal rearrangement of IGH FR2-JH **(G)** and IGK Vκ-Jκ **(H)**.

**Figure 4:**
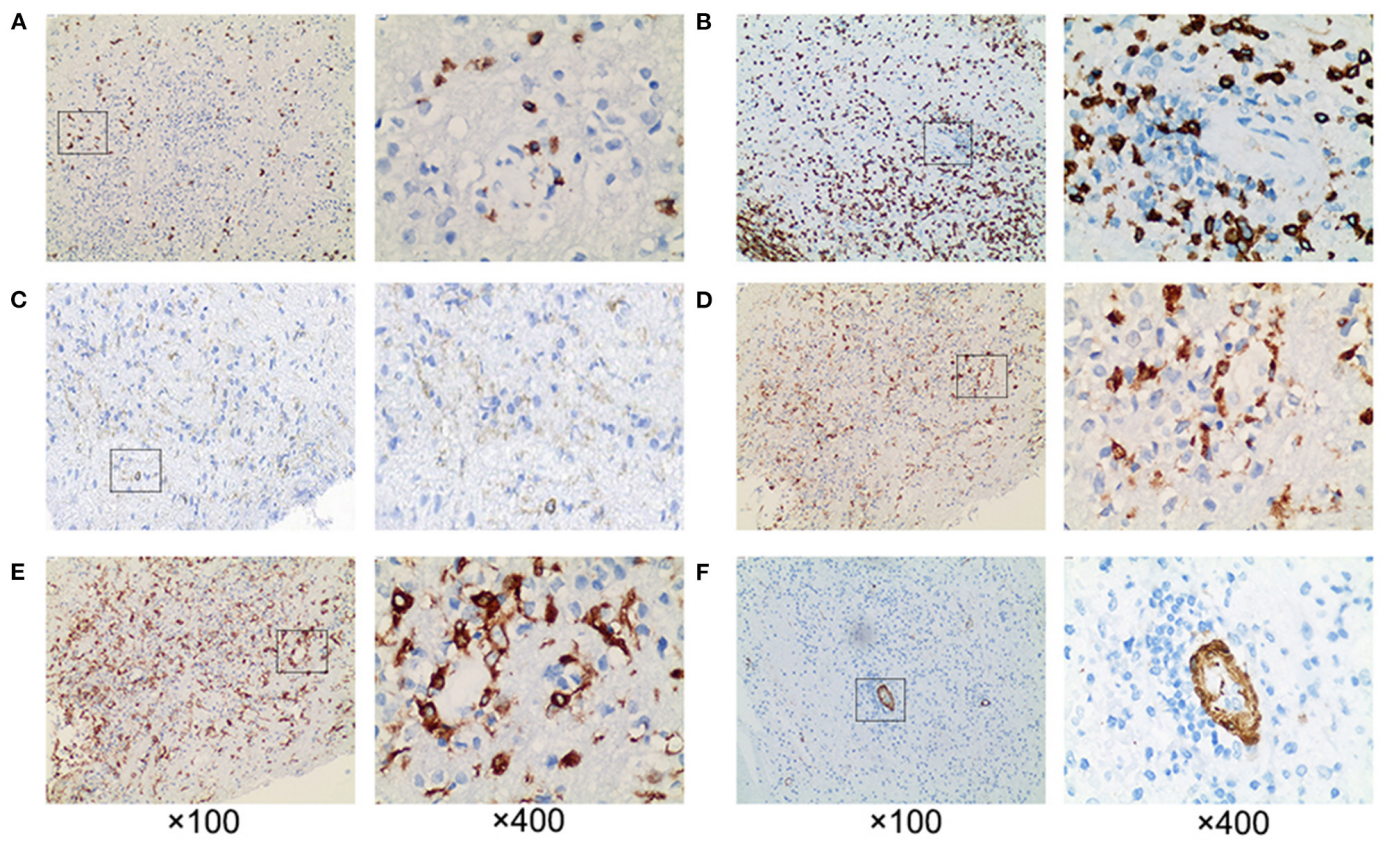
Pathological examination revealed extensive perivascular inflammation. **(A)** CD8+ T cells and **(B)** CD3+ T cells were infiltrated throughout the brain parenchyma, rather than **(C)** CD4+ T cells. Prominent perivascular cuffing of **(D)** CD68+ and **(E)** CD163+ macrophages were seen scattered around small vessels and in the parenchyma. **(F)** The vessel wall indicated by SMA was intact. ×100 and ×400.

Histopathology was confirmatory for diffuse large B-cell lymphoma, non-germinal center B-cell type. Pathological examinations revealed cellular infiltrates of large cells with prominent nucleoli. The tumor was immunopositive for CD20, BCL-2, and MUM-1, with Ki-67 being approximately 80% (Figure 3). CD10 was expressed in only a minority (<10%) of cases. BCL6 protein was expressed in 30% of cases. PCR analysis of B-cell immunoglobulin gene rearrangement detected monoclonal rearrangement of IGH FR2-JH and IGK Vk-Jk (Figure 3). Pathological examination also revealed extensive infiltrating CD3+ T-cells and CD8+ T-cells rather than CD4+ T-cells in the brain parenchyma. The infiltrations of CD68+ and CD163+ macrophages were also observed in the brain parenchyma, which was consistent with GFAP astrocytopathy reported previously (6, 7) (Figure 4).

The patient was transferred to the department of oncology for further treatment. She received a chemotherapy regimen of rituximab plus methotrexate every 21 days. After three courses of chemotherapy, her drowsiness and cognitive impairment significantly improved. Follow-up brain MRI showed resolution of abnormal signal foci in the splenium of the corpus callosum, and lesions in the bilateral basal ganglia and around the third ventricle significantly reduced with complete resolution of the enhancing lesions (Figure 2D). At present, she can complete simple daily activities, such as eating, combing her hair, and dressing.

## Discussion

Glial fibrillary acidic protein is an intracellular protein of the astrocytic cytoskeleton. So far, GFAP antibody detected in serum or CSF has been reported in various disorders such as traumatic brain injury, some neoplasms, autism, Tourette syndrome, multiple sclerosis, diabetes, inflammatory brain disorders following daclizumab treatment, and idiopathic intracranial hypertension, etc. (4, 8–14). As GFAP is a cytoplasmic protein, the pathogenicity of GFAP antibody remains controversial. Perhaps it is only a biomarker for the process of immune inflammation caused by GFAP peptide-specific CD8+ T lymphocytes (2, 15). Some researchers bring forward the question whether GFAP antibody is a secondary phenomenon of reactive gliosis (13). An alternative explanation is that GFAP antibody may be accompanied by an as yet unidentified pathogenic autoantibody targeting the astrocytic plasma membrane (15).

Glial fibrillary acidic protein astrocytopathy is a recently defined autoimmune disease of the central nervous system associated with GFAP-IgG antibody (15). Approximately one-third of patients with GFAP astrocytopathy is accompanied with systemic neoplasia (15). Currently, there are no uniform diagnostic criteria or consensus for GFAP astrocytopathy. Based on relevant literature reports, the key points of diagnosis mainly include the followings (2, 16, 17): (1) Typical clinical presentations include acute or subacute meningitis, encephalitis or myelitis, or any combination of these. (2) Radiological hallmark is a radial periventricular enhancement or T2-hyperintensity, while spinal imaging demonstrates longitudinally extensive lesions with central cord enhancement on MRI. (3) Positive GFAP antibody in CSF (cell-based assay or tissue-based assay). (4) Brain biopsy reveals inflammation around small vessels with vascular wall unaffected. (5) The responsiveness to corticosteroids. (6) Other possible diseases are excluded.

The diagnosis of GFAP astrocytopathy should not be based solely on the positivity of GFAP antibody, especially when the imaging is not typical and the effect of corticosteroid treatment is not satisfactory. Other mimic disorders should be excluded when diagnosing GFAP astrocytopathy. For our patient, the positive presence of GFAP-IgG in CSF suggested GFAP astrocytopathy, but the imaging and treatment response were not typical. Herein, instead of the characteristic MRI feature of brain linear perivascular radial gadolinium enhancement in the white matter perpendicular to the ventricle, our patient had MRI features suggestive of reversible splenial lesion syndrome and multiple abnormal enhancement lesions in bilateral basal ganglia and around the third ventricle. It's suggested that reversible splenial lesions could be considered as a radiological feature in patients with autoimmune GFAP astrocytopathy (18). However, reversible splenial lesions have been reported in patients with heterogeneous pathogenesis triggered by viral infection, hypoglycemia, seizure, lymphoma, and other causes (19–21). Supposedly, a subsided splenial lesion could form either by direct lymphoma involvement or secondarily through the influx of infammatory T-cells (21). As corticosteroids may induce a rapid improvement in clinical symptoms and radiographic features in PCNSL (22), the reversible splenial lesion could also be the MRI presentation of PCNSL in our case. The outcome of our patient suggested that if there was no typical change of radial periventricular enhancement, other diagnoses should be considered even if there is reversible splenial lesion syndrome. Corticosteroid-responsiveness is a hallmark of autoimmune GFAP astrocytopathy (14). Most of the patients had favorable corticosteroid response without relapse (5). This patient demonstrated worsening of clinical conditions despite treatment with corticosteroids during the disease course, which suggested that there may be coexistent malignancy. Finally PCNSL was diagnosed after stereotactic biopsy of a brain lesion. After receiving chemotherapy with rituximab and methotrexate, the clinical symptoms and imaging features of the patient significantly improved.

In addition to the pathological manifestations of lymphoma, the pathology of this patient also conformed to the manifestations of GFAP astrocytopathy. There were perivascular inflammation, T and B cell infiltration, with vascular wall unaffected. CD8+ T cells were frequently found adjacent to neurons and astrocytes, indicating a pathogenic role of CD8+ T lymphocytes in GFAP astrocytopathy, consistent with the pathological features of GFAP astrocytopathy reported previously (23, 24). It is currently not clear whether such inflammatory pathological characteristics reflected a coexistence of lymphoma with GFAP astrocytopathy, or was a genuine feature of PCNSL which can be induced as a consequence of immunosupressive treatment (22)? However, GFAP antibody in CSF of this patient turned negative after steroid treatment, suggesting that the two diseases may coexist, that was, this patient's GFAP astrocytopathy could be the result of a paraneoplastic syndrome. Interestingly, it's reported recently that GFAP astrocytopathy could resemble isolated central nervous system lymphomatoid granulomatosis, which is classified as a subclass of mature B-cell neoplasms (7, 25). Speculatively, there may be a link between lymphomatoid granulomatosis and GFAP astrocytopathy, as well as between PCNSL and GFAP astrocytopathy.

To the best of our knowledge, this is the first case of overlapping syndrome of GFAP astrocytopathy and PCNSL. Primary central nervous system lymphoma is an uncommon and aggressive subtype of extra-nodal non-Hodgkin lymphoma. Most common intracranial lesions on MRI include periventricular white matter, basal ganglia, and corpus callosum. Primary central nervous system lymphoma lesions are often isointense to hyperintense on T2-weighted MRI images and enhanced homogeneously. Corticosteroids cause transient regression of PCNSL at the radiological and histological level. The differential diagnosis includes high grade gliomas, tumefactive demyelinating lesions, metastases, and infectious and granulomatous diseases (22). Confirmation of PCNSL requires a histological or cytological diagnosis (26). For this patient, a diagnosis of PCNSL was supported by histological examination and polymerase chain reaction for B-cell immunoglobulin gene rearrangements.

In conclusion, we should treat the positivity of GFAP antibody rationally, especially seropositive cases. When diagnosing GFAP astrocytopathy, patients need to be investigated for the possibility of other autoimmune conditions and malignancies, which can coexist (16). Cancer screening appropriate for age, sex, and risk factors is recommended for GFAP-specific IgG-positive patients, especially for patients with atypical symptoms. When a PCNSL is considered to be differentiated, early biopsy should be performed before administering a steroid. Prompt diagnosis and initiation of treatment are vital for improving clinical outcomes of patients with PCNSL (27).

## Data Availability Statement

The original contributions presented in the study are included in the article/supplementary material, further inquiries can be directed to the corresponding author/s.

## Ethics Statement

The studies involving human participants were reviewed and approved by Ethical Committee of Second Xiangya Hospital. Written informed consent was obtained from the patient for the publication of any potentially identifiable images or data included in the article.

## Author Contributions

WL designed the study, edited, and revised manuscript. JF and ZT collected data and performed analyses. JF drafted the manuscript. All authors contributed to the article and approved the submitted version.

## Funding

This study was funded by the Scientific Research Fund Project of Hunan Provincial Health Commission (No. 202103070755).

## Conflict of Interest

The authors declare that the research was conducted in the absence of any commercial or financial relationships that could be construed as a potential conflict of interest.

## Publisher's Note

All claims expressed in this article are solely those of the authors and do not necessarily represent those of their affiliated organizations, or those of the publisher, the editors and the reviewers. Any product that may be evaluated in this article, or claim that may be made by its manufacturer, is not guaranteed or endorsed by the publisher.
